# Female Patients with Dermatitis Herpetiformis Show a Reduced Diagnostic Delay and Have Higher Sensitivity Rates at Autoantibody Testing for Celiac Disease

**DOI:** 10.1155/2019/6307035

**Published:** 2019-12-29

**Authors:** Emiliano Antiga, Veronica Bonciolini, Simone Cazzaniga, Mauro Alaibac, Antonino Salvatore Calabrò, Carla Cardinali, Emanuele Cozzani, Angelo Valerio Marzano, Giuseppe Micali, Tarcisio Not, Pietro Quaglino, Camilla Vassallo, Luigi Naldi, Marzia Caproni, the GISED Group and the Italian Group for Cutaneous Immunopathology

**Affiliations:** ^1^Department of Health Sciences, Section of Dermatology, University of Florence, Florence, Italy; ^2^Centro Studi GISED, Bergamo, Italy; ^3^Dermatology Department, Inselspital University Hospital, Bern, Switzerland; ^4^Unit of Dermatology, Department of Medicine, University of Padua, Padua, Italy; ^5^Department of Experimental and Clinical Biomedical Sciences, Gastroenterology Unit, University of Florence, Florence, Italy; ^6^Operative Unit of Dermatology, Azienda USL Toscana Centro, Prato, Italy; ^7^Section of Dermatology, University of Genoa, Di.S.Sal., San Martino Policlinic Hospital, Genoa, Italy; ^8^Dermatology Unit, IRCCS Cà Granda Foundation, Department of Pathophysiology and Transplantation, University of Milan, Milan, Italy; ^9^Dermatology Clinic, University of Catania, Catania, Italy; ^10^Institute for Maternal and Child Health-IRCCS “Burlo Garofolo” Trieste, Trieste, Italy; ^11^Department of Medical Sciences, Section of Dermatology, University of Turin, Turin, Italy; ^12^Department of Clinical-Surgical, Diagnostic and Pediatric Science, Institute of Dermatology, University of Pavia, Pavia, Italy; ^13^Department of Dermatology, AULSS8 Berica, Ospedale San Bortolo, Vicenza, Italy; ^14^GISED (Italian Group for Epidemiological Studies in Dermatology) Group and Italian Group for Cutaneous Immunopathology, Italy

## Abstract

**Objective:**

Our objective was to characterize the demographic information, clinical features, and laboratory data of patients with dermatitis herpetiformis (DH).

**Methods:**

In this multicentre cross-sectional study, consecutive patients with a new diagnosis of DH that referred to nine different Italian centers between 2011 and 2016 were characterized assessing demographic, clinical and laboratory findings, and evaluating gender and age differences across selected variables.

**Results:**

A total of 151 patients were included. Among them, 81 (53.6%) were males and 70 (46.4%) were females, with a male to female ratio of 1.2 : 1. The median age at the time of diagnosis was 41 years (range 0–85). Males had a significant longer diagnostic delay if compared to females (9 vs. 3 months, respectively; *p* = 0.01). Direct immunofluorescence was positive in 94.7% of the patients, while duodenal biopsy showed partial to total villous atrophy in 70.1% of patients. All the females resulted positive to at least one of the antibodies tested, while a total of 12 male patients (10.5%) tested negative to celiac-specific antibodies. Female patients had a high rate (14.1%) of autoimmune thyroiditis.

**Conclusions:**

Our study confirmed some of the most relevant data regarding DH that have been previously reported in the literature. In addition, we found a reduced diagnostic delay in females with respect to males, possibly related to the higher sensitivity of serologic testing in females with DH compared to males. Finally, we demonstrated that intestinal involvement could be severe in patients with DH and that females should be tested for thyroiditis.

## 1. Introduction

Dermatitis herpetiformis (DH) is a chronic inflammatory skin disease that is considered the specific cutaneous manifestation of celiac disease (CD) [1]. However, only a minority of celiac patients develop DH. Moreover, while the prevalence of CD in the population is about 1-2% with increasing incidence, recent studies demonstrated a reduction of the incidence of DH in the last decades, with figures of less than 3-4 cases per 100 000 inhabitants, making DH a rare disease [2].

DH has a polymorphic clinical presentation, showing erythema, papules, wheals, vesicles, pustules, or blisters often in typical sites, like the elbows, knees, and sacral areas but also in a typical sites, such as the folds, the palmo-plantar regions, and the scalp, which in some cases may represent the only affected sites [3]. Itch is usually very severe, and the patients may present with scratching lesions alone.

Histopathology of the skin may show some typical signs but is usually unspecific; by contrast, the gold standard for the diagnosis is the finding of granular immunoglobulin (Ig) A deposits at the dermal papillae or along the dermal–epidermal junction by direct immunofluorescence (DIF) of perilesional skin, that is not always available and may provide false-negative results [4].

Due to its rarity, its clinical heterogeneity, the low specificity of histopathology and the methodological issues of DIF, the diagnosis of DH is often difficult, with a delay in the introduction of GFD [5]. As a consequence, a better knowledge of the disease is paramount in order to improve the management of the patients, considering that DH is not only a mere skin disease but can be associated with all the complications and risks related to CD [6].

Therefore, we performed a multicenter epidemiological study on DH conducted at several dermatologic outpatient clinics in Italy, with the aim to characterize the demographic information, the clinical features, and the laboratory data of the patients.

## 2. Methods

This was a multicenter cross-sectional study investigating consecutive patients with a new diagnosis of DH that referred to the Units of Florence, Milan, Padua, Trieste, Genoa, Prato, Turin, Catania, and Pavia between 2011 and 2016. The study was conducted according to the statements of the Declaration of Helsinki and was approved by the institutional review board of each hospital involved in the study; all the patients provided written informed consent.

Demographic, clinical, and laboratory findings were collected from each patient. Among them, data on the morphology and distribution of the skin lesions, on the intensity of pruritus using a visual analogue scale (VAS) ranging from 1 to 10, on specific serology (EMA, anti-tTG, antideamydated gliadin peptides (DGP); antiepidermal transglutaminase antibodies (eTG), that are considered to have a high sensitivity and specificity for DH, were not included in the analysis because they were tested only in a minority of patients since they are not routinely investigated), as well as on cutaneous or systemic associated diseases were reported when available.

Moreover, data on immunopathological findings were collected at the time of the diagnosis, with patients still on normal gluten-containing diet. In particular, duodenum biopsies, performed on about half of the patients, were assessed for the severity of CD using the Marsh classification modified by Oberhuber [7].

Finally, all the findings of DIF were reported, including the type, the localization, and the morphology of the immune deposits.

### 2.1. Statistical Analysis

For descriptive purpose continuous data were presented as medians with ranges, while categorical variables as numbers with percentages. Gender and age differences across selected variables were assessed by means of Mann–Whitney *U* test and Pearson's *Χ*^2^ test (or Fisher's exact test where required) for continuous and categorical data, respectively. All tests were considered statistically significant at *p*-value < 0.05. Analyses were performed with SPSS v.20.0 (IBM Corp, Armonk, NY, US).

## 3. Results

### 3.1. Demographic Data

**Figure 1:**
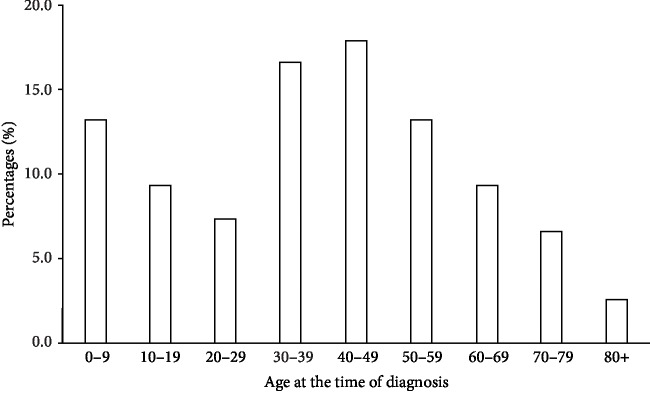
Histogram of age distribution at the time of diagnosis among patients included in the study.

During the 5-year study period, in the 9 Dermatology Units participating to the study, a total of 151 patients were included. Among them, 81 (53.6%) were males and 70 (46.4%) were females, with a male to female ratio of 1.2 : 1. The median age at the time of diagnosis was 41 years (range 0–85), with no differences between males and females (*p* = 0.68). Thirty-one patients (21.4%) were under the age of 18 at the time of diagnosis, while 23 (15.9%) were aged 65 years or above, with no differences in the distribution of females and males. There were two peaks in the distribution of age at the time of diagnosis, one in the first decade and the other in the fourth-to-sixth decades (Figure 1).

The median diagnostic delay in months calculated as the difference between the age at diagnosis and the age at which the first symptoms and signs of DH occurred as reported by the patients was of 6 months (range 0–239); interestingly, males had a significant longer diagnostic delay if compared to females (9 vs. 3 months, respectively; *p* = 0.01).

### 3.2. Clinical Features

The majority of patients had the classical clinical features of DH, showing a polymorphic skin eruption consisting of erythema, papules, and vesicles, that were found in 111 (73.5%), 92 (60.9%), and 98 (64.9%) patients, respectively. Only few patients showed wheals (12.6%) or bullous lesions (8.6%).

The distribution of the lesions was typical in most of the patients, being the elbows the most frequently involved area (124 patients, 84.1%). Other commonly involved sites were the sacral region (82 patients, 54.3%), the knees (74 patients, 49%) and the shoulders (40 patients, 26.5%). The face was affected in 14.6% of patients, with a predominance in females than in males, although without statistical significance (19.7% vs. 10.1%, respectively; *p* = 0.10). Only 3 patients had mucosal lesions.

Some clinical differences were found between paediatric and elderly patients. In particular, blisters were detected in the latter but not in the former (22.7% vs. 0%; *p* = 0.009); by contrast, shoulders were significantly more involved in paediatric patients than in patients above the age of 65 (61.3% and 13%, respectively; *p* < 0.001).

The majority of patients had scratching lesions (75.5%) and reported pruritus (93.3%), with a median VAS of 6. About a third of the patients (29.5%) had severe pruritus with a VAS ≥ 8. No correlation was found between VAS and diagnostic delay of the patients.

Interestingly, paediatric patients had significantly less pruritus than the elderly ones (median VAS 4 and 7, respectively; *p* = 0.02). Accordingly, patients above the age of 65 showed scratching lesions more frequently than those below the age of 18 (90.9% and 48.4%, respectively; *p* = 0.001).

### 3.3. Direct Immunofluorescence and Histopathological Findings

DIF findings were positive in 143 out of 151 patients (94.7%), showing the pathognomonic finding of granular IgA deposits (Table 1). Moreover, for 120 patients, the description of the immune deposits was more accurate and reported the presence of other immunoreactants, accordingly, 5.6% of the patients showed IgG deposits, 39.1% IgM deposits, and 68% C3 deposits at the dermal papillae or along the dermal-epidermal junction in a DH-like pattern. By contrast, perivascular IgA deposits in the superficial dermis were found only in 8.7% of patients.

A detailed description of histopathological findings was available for review in 74 patients. In the majority of cases, typical histopathological findings were detected, including the presence of subepidermal blistering (75.7%) as well as inflammatory infiltration at perivascular areas of the superficial dermis (95.6%) and at the dermal papillae (67.6%). Inflammatory cells consisted of neutrophils (93.3%); lymphomonocytes (91.1%), and eosinophils (85.7%).

Duodenal biopsies were available for 77 patients (Table 2). Among them, 6 patients (7.8%) showed a normal mucosa,11 patients (14.3%) showed a Marsh 1 degree, indicating the presence of an increased number of intraepithelial lymphocytes, 6 patients (7.8%) showed a Marsh 2 degree indicating the presence of an increased number of intraepithelial lymphocytes and of crypt hyperplasia; 54 patients (70.1%) showed a Marsh 3 degree, indicating the presence of an increased number of intraepithelial lymphocytes and of crypt hyperplasia together with partial to total villous atrophy. Among the latter, 15 patients (21.1%) had a Marsh 3a degree, indicating mild villous atrophy, 26 (36.6%) had a Marsh 3b degree, indicating subtotal villous atrophy, and 13 (18.3%) had a Marsh 3c degree, indicating total villous atrophy. No correlation was found between the intestinal involvement and diagnostic delay or other clinical features of the patients.

### 3.4. Serologic Findings

Serologic investigation assessing CD-specific autoantibodies was performed in most of the patients (Table 1). None of the patients tested had IgA deficiency. Anti-tTG IgA antibodies were proven to be the most sensitive serologic markers for DH, being positive in 101 out of 110 (91.8%) of the patients. Moreover, 80 out of 93 patients tested for EMA antibodies (86%) resulted positive; similar results were found for IgG anti-DGP autoantibodies, that resulted positive in 45 out of 53 of the patients tested (84.9%), while only 28 out of 53 patients (52.8%) tested positive for IgA anti-DGP autoantibodies. Interestingly, females showed positivity to IgG anti-DGP autoantibodies significantly more frequently than males (100% and 75.8%, respectively, *p* = 0.02). In addition, all females resulted positive to at least one of the antibodies tested, while a total of 12 male patients (10.5%) tested negative to all the serologic tests performed (seronegative DH).

### 3.5. Celiac Disease and Other Associated Diseases

Since DH is the specific skin manifestation of CD, all patients with DH are concomitantly diagnosed also as having CD and start a GFD. However, in our case series, a diagnosis of CD prior that of DH was made in 30 patients (19.9%).

Associated skin diseases were diagnosed in 23 out of 151 patients with DH (15.2%). Among them, psoriasis (3.3%), atopic dermatitis (2.7%) and vitiligo (2%) were the most frequently associated skin diseases (Table 3).

Associated systemic diseases were found in 43 out of 151 patients with DH (28.5%). Among them, autoimmune thyroiditis was the most frequent, and was found in 11 patients (7.3%). Interestingly, the prevalence of autoimmune thyroiditis was significantly higher in females than in males with DH (14.1% vs. 1.3%, respectively; *p* = 0.003) (Table 3).

## 4. Discussion

In the present study, we reported the epidemiological, clinical, and immunopathological data of an Italian multicenter retrospective study on patients with DH. DH is a rare disease with an incidence that varies between different geographical areas. While it is relatively more common in Northern Europe [2, 8] and in the US [9], DH is very rare in almost all the other countries, being exceptional in the Far East, although some case series of patients with DH were previously reported in the literature [10], especially from Japan [11, 12]. In Italy, the exact incidence of DH is not known, although it is considered a relatively high incidence country [13]. This was confirmed by our work, which in a period of 5 years was able to collect 151 patients with DH, being one of the largest case series studies on the disease.

While a female prevalence is reported in CD, previous studies on DH showed a male to female ratio ranging from 1.1 to 1.9 [2]. Accordingly, our study confirmed such data, reporting a male to female ratio of 1.2 : 1. This may be possibly related to the fact that male patients are seronegative for CD more frequently than females that in turn may favour the longer diagnostic delay in such population, allowing more time for DH to develop [2].

Regarding the age at the diagnosis, we confirmed the data from previous studies [2, 10, 14, 15] showing a median age of 41, without differences between males and females. By contrast, we found a high number of DH patients under the age of 18 (21.4%), while in other case series pediatric population represented only a minor proportion of patients with DH [2]. A possible explanation for this discrepancy may be represented by the recruitment of pediatric patients; some of the Dermatology centers involved in this study usually see also pediatric patients with DH, increasing the number of patients below the age of 18. Another explanation might be related to different genetic background or different exposure to gluten; accordingly, a previous study from our group reported a high percentage of pediatric patients among those diagnosed with DH [13].

Of note, the median delay between the onset of symptoms and the diagnosis was of 6 months and therefore similar to that of a large Finnish study (10 months, decreased to 8 months in the study period 2000–2014)[5] but shorter than in other studies, such as in a study from China [10], where DH is rarer than in Italy and the median diagnostic delay was found to be of 44 months. This may be related to the focus on DH of the centers involved in this study, as well as to the awareness on gluten-related disorders in Italy. Interestingly, females had a significantly shorter diagnostic delay than males, probably due to a major attention of the former to their skin symptoms and to the higher frequency of seronegative patients in the latter. While this finding was confirmed by some studies, suggesting that diagnostic delay may explain the higher incidence of DH in males [2], other papers reported a higher diagnostic delay in females [5].

From a clinical point of view, our study confirmed what were previously known on DH about the morphology of the lesions, their distribution and the occurrence of pruritus that was moderate to severe in the majority of the patients [16]. Itching is one of the major concerns for patients with DH, and it is related at least in part to the prominent T helper 2 phenotype of the immune response with release of several cytokines such as IL-31 [17].

Interestingly, our data showed that elderly patients had significantly more severe itching and a higher frequency of scratching lesions than pediatric ones. These results could be explained, at least partly, by the higher degree of dryness of elderly skin; moreover, a higher frequency of blisters was observed in patients above the age of 65 that might reflect a more severe inflammatory disease.

Although no statistical differences were found in lesion morphology between males and females, the latter showed a higher frequency of face involvement, being the only affected area in one female patient of our series. In the literature, face involvement is not infrequent; however, it has been reported as the sole affected site only in four cases [18].

The gold standard for the diagnosis of DH is the presence of granular IgA deposits at the papillary tips or along the dermal-epidermal junction found by DIF. In our case series, 94.7% of the patients showed IgA deposits, while the other 5.3% did not. The latter, however, were diagnosed as having DH due to suggestive clinical findings, compatible histopathological examination, typical serology for CD, bowel biopsy positive for CD, and clinical response to a GFD. The percentage of DIF-negative cases are is in agreement with previous studies, due to the sensitivity of DIF that is less than 100% [19]. False-negative results may occur in several instances, including the wrong selection of the biopsy site (if performed in involved skin) or if the patient is on GFD (that was the case of 2 out of our 8 DIF-negative patients).

According to the guidelines for CD [20], a patient receiving the diagnosis of DH is automatically considered as having CD and does not need a bowel biopsy. However, in our case series, more than half of the patients had intestinal histopathological examination; among them, 7.8% showed negative results. These findings are in agreement with previous data from literature, where bowel biopsy has been demonstrated to provide false- negative results in up to 10% of the patients with CD, depending on the number of duodenal specimens that are investigated [21]. Moreover, we found that more than 70% of the patients had histopathological involvement of the duodenum with a Marsh 3 degree, while more than 50% of the patients had subtotal or total villous atrophy. Although recent papers showed a lower incidence of severe villous atrophy in patients with DH than in CD, with a reduction in the last years [22, 23], our finding is in agreement with previous studies from the literature [24].

In general, serological findings in our patients were similar to those from other DH case-series reported in the literature as well as those of CD patients, with anti-tTG antibodies being the most sensitive marker for the diagnosis of DH [3]. In addition, we found a high positive rate for EMA, which was higher than that of anti-DGP antibodies that, in CD, are considered more sensitive than EMA [25]. Unfortunately, anti-eTG antibodies were investigated only in a minority of the patients, since they are not routinely performed by standard laboratories and, therefore, were excluded from our analysis.

An interesting finding of our study was the higher positive rate of antibody testing in females. This difference was statistically significant only for IgG anti-DGP antibodies; however, none of the female patients in our study were completely seronegative, while 10.5% of the male patients did not show any CD-specific circulating autoantibody. Previous studies showed similar results, with serologic testing being less sensitive for male patients with CD [26]; such a finding might explain the higher diagnostic delay in males than in females.

Finally, in our cohort, few patients had associated skin or systemic diseases; among them, autoimmune thyroiditis was the most frequent and, according to the literature, it was more prevalent in females, with a prevalence that was higher than that of the Italian general population [27]. Therefore, thyroid function and autoantibodies should be tested in each female patient with DH.

In conclusion, our study confirmed some of the most relevant data regarding DH that were previously reported in the literature, investigating a high number of cases from Italy, where such data were scarce. In addition, some interesting points arise from our study. Females had a reduced diagnostic delay with respect to males, possibly related to the higher sensitivity at serologic testing in them. Moreover, at variance with previous believes, we demonstrated that intestinal involvement could be severe in patients with DH, with even severe villous atrophy at the duodenal biopsy. Finally, we recommend screening female DH patients for autoimmune thyroiditis.

## Figures and Tables

**Table 1:** Direct immunofluorescence results and circulating autoantibodies found in patients with dermatitis herpetiformis at the time of the diagnosis.

		Sex				Total		*P*-value^∗^
		M		F				
		*N*	%	*N*	%	*N*	%	
DIF	−	4	6.1%	4	6.5%	8	5.3%	1
	+	77	95.1%	66	94.3%	143	94.7%	
Anti-tTG IgA	−	6	11.3%	2	3.6%	9	8.2%	0.15
	+	47	88.7%	54	96.4%	101	91.8%	
EMA IgA	−	9	18.4%	3	7.0%	13	14.0%	0.11
	+	40	81.6%	40	93.0%	80	86.0%	
Anti-DGP IgG	−	8	24.2%	0	0.0%	8	15.1%	**0.02**
	+	25	75.8%	20	100.0%	45	84.9%	
Anti-DGP IgA	−	17	51.5%	8	40.0%	25	47.2%	0.21
	+	16	48.5%	12	60.0%	28	52.8%	

DIF: direct immunofluorescence; tTG: tissue transglutaminase; EMA: endomysium antibodies; DGP: deamydated gliadin peptides.^∗^Pearson's *Χ*^2^ test or Fisher's exact test where required.

**Table 2:** Intestinal involvement in patients underwent duodenal biopsy based on the Marsh classification modified by Oberhuber [8].

		Sex				Total		*P*-value^∗^
		M		F				
		*N* = 33	%	*N* = 38	%	*N* = 71	%	
Grade	1	5	15.2%	6	15.8%	11	15.5%	0.81
	2	4	12.1%	2	5.3%	6	8.5%		
	3a	8	24.2%	7	18.4%	15	21.1%		
	3b	11	33.3%	15	39.5%	26	36.6%		
	3c	5	15.2%	8	21.1%	13	18.3%		

^∗^Fisher's exact test where required.

**Table 3:** Skin and systemic associated diseases found in patients with dermatitis herpetiformis.

Skin associated diseases	No (%)	Systemic associated diseases	No (%)
Psoriasis	5 (3.3)	Autoimmune thyroiditis	11 (7.3)
Atopic dermatitis	4 (2.7)	Arterial hypertension	9 (6.0)
Vitiligo	3 (2.0)	Type 2 diabetes mellitus	7 (4.6)
Urticaria	2 (1.3)	Osteoporosis	5 (3.3)
Contact eczema	2 (1.3)	Allergic rhinoconjunctivitis	3 (2.0)
Rosacea	2 (1.3)	Autoimmune gastritis	1 (0.7)
Telogen effluvium	2 (1.3)	Crohn's disease	1 (0.7)
Bullous pemphigoid	1 (0.7)	Gastric cancer	1 (0.7)
Pityriasis rosea gibert	1 (0.7)	Hairy cell leukemia	1 (0.7)
Prurigo nodularis	1 (0.7)	Prostatitis	1 (0.7)
		Rheumatoid arthritis	1 (0.7)
		Beta thalassemia	1 (0.7)
		Type 2 diabetes mellitus	1 (0.7)

## Data Availability

The data used to support the findings of this study are available from the corresponding upon request.

## Additional Points

*Key Points*. Female patients with dermatitis herpetiformis had a reduced diagnostic delay with respect to males, possibly related to the higher sensitivity of serologic testing in them. Intestinal involvement could be severe in patients with dermatitis herpetiformis, with even severe villous atrophy at the duodenal biopsy. Screening for autoimmune thyroiditis is recommended in female patients with dermatitis herpetiformis.
